# Toward ultrafast magnetic depth profiling using time-resolved x-ray resonant magnetic reflectivity

**DOI:** 10.1063/4.0000109

**Published:** 2021-06-23

**Authors:** Valentin Chardonnet, Marcel Hennes, Romain Jarrier, Renaud Delaunay, Nicolas Jaouen, Marion Kuhlmann, Nagitha Ekanayake, Cyril Léveillé, Clemens von Korff Schmising, Daniel Schick, Kelvin Yao, Xuan Liu, Gheorghe S. Chiuzbăian, Jan Lüning, Boris Vodungbo, Emmanuelle Jal

**Affiliations:** 1 Sorbonne Université, CNRS, Laboratoire de Chimie Physique-Matière et Rayonnement, 75005 Paris, France; 2 Synchrotron SOLEIL, L'Orme des Merisiers, Saint-Aubin, B.P. 48, 91192 Gif-sur-Yvette, France; 3 Deutsches Elektronen-Synchrotron, 22607 Hamburg, Germany; 4 Max Born Institut für Nichtlineare Optik und Kurzzeitspektroskopie, 12489 Berlin, Germany; 5 Helmholtz-Zentrum Berlin für Materialien und Energie, 14109 Berlin, Germany

## Abstract

During the last two decades, a variety of models have been developed to explain the ultrafast quenching of magnetization following femtosecond optical excitation. These models can be classified into two broad categories, relying either on a local or a non-local transfer of angular momentum. The acquisition of the magnetic depth profiles with femtosecond resolution, using time-resolved x-ray resonant magnetic reflectivity, can distinguish local and non-local effects. Here, we demonstrate the feasibility of this technique in a pump–probe geometry using a custom-built reflectometer at the FLASH2 free-electron laser (FEL). Although FLASH2 is limited to the production of photons with a fundamental wavelength of 4 nm ( ≃310 eV), we were able to probe close to the Fe *L*_3_ edge ( 706.8 eV) of a magnetic thin film employing the third harmonic of the FEL. Our approach allows us to extract structural and magnetic asymmetry signals revealing two dynamics on different time scales which underpin a non-homogeneous loss of magnetization and a significant dilation of 2 Å of the layer thickness followed by oscillations. Future analysis of the data will pave the way to a full quantitative description of the transient magnetic depth profile combining femtosecond with nanometer resolution, which will provide further insight into the microscopic mechanisms underlying ultrafast demagnetization.

## I. INTRODUCTION

Ultrashort optical excitations generate highly out-of-equilibrium states in ferromagnetic systems. Hot electrons are created upon absorption of the radiation and induce changes in the magnetic anisotropy and the crystal field which couple and become time-dependent.^1^ It was in 1996 that Beaurepaire *et al.* measured ultrafast demagnetization in a ferromagnetic Ni thin film on sub-picosecond timescales.^2^ This discovery opened up a new field of research: femtomagnetism, which aims at understanding the impact of ultrashort light pulses on magnetic systems.^1^ However, after more than 20 years of research, the microscopic mechanisms at play during ultrafast demagnetization still remain poorly understood.^3,4^ In addition to this fundamental interest, experiments performed since then have demonstrated that this phenomenon is key for applications of magnetization control on the femtosecond timescale, as illustrated by the demonstration of all-optical magnetization reversal^5^ and spin current propagation.^6^

In the quest for a consensus concerning the microscopic basis underlying ultrafast demagnetization, two models have been intensively discussed by the community: (i) an Elliot–Yafet-like mechanism,^7^ where spin flips result from phonon scattering and (ii) a superdiffusive model,^8^ where spin angular momentum is transported out of the magnetic system by polarized spin currents. These two models can be extended to two larger categories: local and non-local phenomena.^9,10^

Distinguishing between these two channels for the removal of spin angular momentum is notoriously difficult and calls for methods that associate state-of-the-art time and spatial resolution to element selectivity. Despite the advent of x ray and extreme ultraviolet (XUV) free-electron lasers (FELs) as well as of high harmonic generation sources,^11,12^ achieving nanometer spatial resolution remains a challenge. One way to overcome this technical hurdle is to study spin-valve structures and use an element selective probe to detect if superdiffusive spin currents travel or not from one magnetic layer to the other.^13,14^ A path toward higher spatial resolution is also illustrated by Kerr and Faraday magneto-optical measurements performed on both the front and back sides of simple ferromagnetic layers^9,15^ or of spin-valve structures.^16^ A further way to achieve higher spatial resolution is to monitor interfaces by magnetization-induced second harmonic generation.^17,18^ The latter approach is only sensitive to the interface, which causes the inversion symmetry breaking.^19^ In order to probe entire magnetic layers with high spatial resolution, small-angle x-ray scattering was performed on magnetic thin films presenting magnetic domains. This technique allows for a good in-plane spatial resolution; however, the results obtained under various conditions (different pump types and sample compositions) by several groups yielded contradictory conclusions.^20–24^ Further experimental and theoretical studies suggest that local and non-local processes can actually coexist but will give rise to very different evolutions of the magnetization in the direction perpendicular to the surface.^25–29^ Therefore, spatial depth resolution is required in order to gain insight into the spin removal process.^25–28^

X-ray resonant magnetic reflectivity (XRMR) is an excellent technique to retrieve the magnetic depth profile of thin films.^30,31^ It has recently been applied in pump–probe geometry to perform time-resolved XRMR (TR-XRMR) and study femtomagnetism.^32,33^ While Gutt *et al.*^33^ have probed a magnetic trilayer film at the Fe $M_{3,2}$ edge, showing a magnetic depth spatial resolution of tens of nanometer, applying this technique at *L* edges of transition metals will allow us to achieve a sub-nanometer magnetic spatial resolution.^30,31^ In our previous work,^32^ we performed a TR-XRMR experiment on a Ni thin film at the Ni *L*_3_ edge. In this study, made at the femtoslicing source of BESSY II, we demonstrated that TR-XRMR experiments allow us to probe simultaneously ultrafast magnetic and structural dynamics, but need high photon flux, such as the one provided by FEL, if one wants to discriminate the different transient depth magnetic profile. However, access to FEL providing soft x rays is limited by the number of such sources.

In this paper, we demonstrate that TR-XRMR can be performed close to the Fe *L*_3_ edge ( 706.8 eV) at the FEL FLASH2. Our approach is based on the use of the 3rd harmonic generation of the FEL radiation, allowing us to work well above the highest possible energy of the first harmonic ( ≈310 eV). We explain how we probed simultaneously magnetic asymmetry and structural signals of a 15 nm Fe thick film, thanks to our custom-built reflectometer. Our measurements, performed at 701±3.5 eV, demonstrate that there are (i) a non-homogeneous loss of magnetization in the first hundreds of femtoseconds and (ii) a thickness oscillation of our thin films, which is induced by a strain wave, at larger time scales (picoseconds). While future quantitative analysis will enable to retrieve the transient depth magnetic and structural profile with sub-nanometer spatial resolution, this article explains the technical challenges of such measurements and paves the way for future experiments aiming at understanding the microscopic foundations of ultrafast magnetization.

## II. PRINCIPLE AND EXPERIMENT

### A. X-ray resonant magnetic reflectivity (XRMR)

**FIG. 1.**
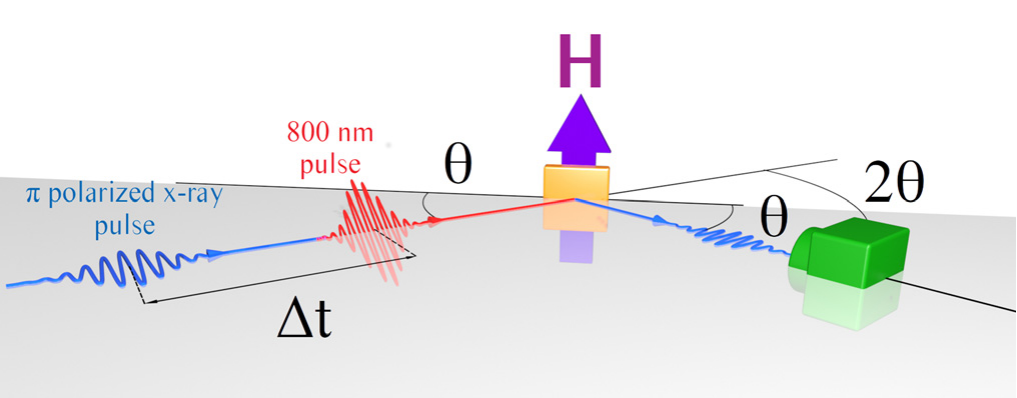
Sketch of the TR-XRMR setup in the transverse configuration with the sample in orange, the x-ray detector in green, the transversal magnetic field H in violet, the x-ray pulse in blue, the infrared pump pulse in red, and the scattering plane in gray. Note that the static XRMR setup is identic without the pump pulse.

XRMR is an experimental approach based on the combination of x-ray reflectivity (XRR) and x-ray magnetic circular dichroism (XMCD).^34^ In XRR, one usually measures the change of the reflected intensity as a function of the incidence angle *θ*. The XRR data can be fitted^35^ within a straightforward matrix formalism^36^ and deliver a variety of parameters such as the density, the thickness, and surface roughness with sub-nanometer resolution,^34,37^ typically of 2 Å. XMCD is the dependence of x-ray absorption as a function of the circular polarization of the incoming x rays as well as their energy, with a strong effect at specific core level edges. It provides element specific information on the magnetic properties of the sample.^38^ When performing XRR measurements with photon energies tuned to resonance with large XMCD contrast, as, for instance, the $L_{3,2}$ resonance of iron, the reflectivity signal becomes a function of the magnetization orientation with respect to the helicity of the incoming radiation. Taking into account the XMCD in the complex refraction index, it is possible to rewrite the matrix formalism to simulate reflectivity data at resonance to retrieve the magnetization profile.^34,39,40^ However, it is worthwhile to mention that very few FEL sources provide circularly polarized light in the soft x-ray regime. In order to circumvent this limitation, it is instructive to write the electric field in the linear polarization base and observe that, depending on the polarization channel, XRMR performed with linearly polarized light is sensitive to the spatial direction of the magnetization. In particular, the *π*–*π* channel is sensitive to the transverse magnetization, as it is the case in transverse magneto-optic Kerr effect experiments.^39^ Here *π* is the common notation for linear polarization lying in the scattering plane (Fig. 1).

In this contribution, we report on measurements performed in the transverse configuration with incoming *π* polarization. The sample, a thin Fe film, was magnetized along the normal to the scattering plane as shown in Fig. 1. Two reflectivity curves, *I*^+^ and *I*^−^, were recorded with opposite magnetic fields indicated here as + and −. Following Jal *et al.*,^32^ two relevant signals can be extracted: (i) the structural signal $S=(I^{+}+I^{-})/2$, which is sensitive to the (apparent) electronic charge density and is independent of the sample magnetization;^41,42^ (ii) the magnetic asymmetry signal $A=(I^{+}-I^{-})/(I^{+}+I^{-})$, which is proportional to the ratio of magnetic to charge contributions.^43^

### B. Sample characteristics

The sample is a polycrystalline ferromagnetic Fe thin film grown by sputter deposition on top of a thermally oxidized Si substrate with additional Pt and Ta buffer layers. A Pt capping layer was used to prevent oxidation, resulting in a Si/ ${\text{Ta}}_{3\;\text{nm}}$/ ${\text{Pt}}_{3\;\text{nm}}$/ ${\text{Fe}}_{15\;\text{nm}}$/ ${\text{Pt}}_{3\;\text{nm}}$ multilayer structure.

Static magneto-optic Kerr effect measurements were used to check the expected in-plane magnetization of the Fe film and showed a square hysteresis loop with a coercive field of 4 mT.

**FIG. 2.**
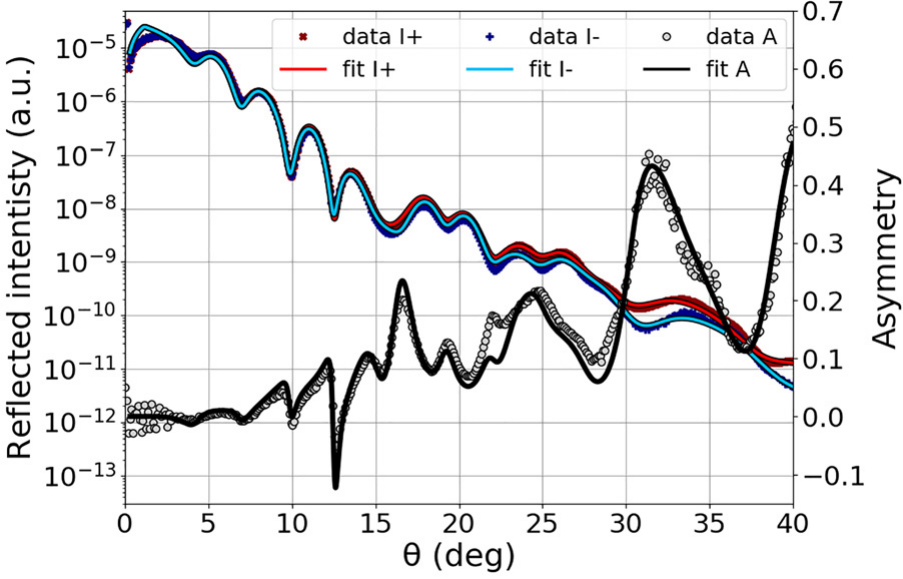
Static x-ray reflectivity curves recorded at Synchrotron SOLEIL at the Fe *L*_3_ edge (704.7 eV; resolving power $\Delta E/E=2\times 10^{-4}$), with *π* incoming polarization, for two opposite in-plane magnetization directions perpendicularly to the scattering plane. The reflectivity data (dots) and fits (lines), for both directions of the magnetic field (+ and −) *I*^+^ and *I*^−^, are shown in red and blue. The derived asymmetry *A* (gray dots) is compared to the simulated data (black line).

The static structural and magnetic parameters of the sample were furthermore characterized by static XRMR measurements at the SEXTANTS^44,45^ beamline of Synchrotron SOLEIL. The angular scan recorded with 704.7 eV photons is shown in Fig. 2 for both applied field directions (red and blue dots), as well as the derived asymmetry (gray dots). Further scans (not shown) were recorded in a non-resonant condition (600 eV) and with energies encompassing the *L*_3_ resonance (see Table I).

**TABLE I.** Average of structural parameters derived from the fits of static XRMR curves obtained at Synchrotron SOLEIL with photon energies tuned to 600.0, 704.7, 705.1, 706.1, 707.1, 708.1, and 709.1 eV as well as from XRR measurements at 8047 eV.

	Density (mol cm^−3^)	Thickness (nm)	Roughness (nm)
Pt	0.097	3.2 ± 0.2	0.9 ± 0.1
Fe	0.120	18.4 ± 0.2	0.8 ± 0.2
Pt	0.097	3.9 ± 0.2	0.3 ± 0.1
Ta	0.078	3.3 ± 0.2	0.5 ± 0.2
Si	0.083		0.3 ± 0.2

From these experimental data, we used a matrix formalism, implemented in DYNA,^34^ to extract the structural and magnetic parameters. All the structural parameters are listed in Table I and were confirmed by XRR recorded with hard x rays [Cu $K_{\alpha_{1}}$ ( ≈8047 eV)]. The reflectivity measurements give thicknesses in good agreement with the nominal parameters set for the sputter deposition, though 10% to 20% larger. This can be explained by the fact that the quartz balance used for the calibration during sample growth tends to slightly underestimate the thicknesses. It is relevant to note that the large angular range covered for the XRMR measurements imposes tight boundary conditions on the fit parameters, thus resulting in small associated uncertainties.

The magnetic asymmetry is, in average, correctly reproduced with a uniform magnetic depth profile through the entire iron layer; however, it is not very good for the angular range [20°,30°]. Note that a more complex model with interface effects can probably correct this disagreement.^31,46^ However, this is beyond the scope of this paper and will be the aim of a future article.

### C. Instrument design for TR-XRMR measurements

**FIG. 3.**
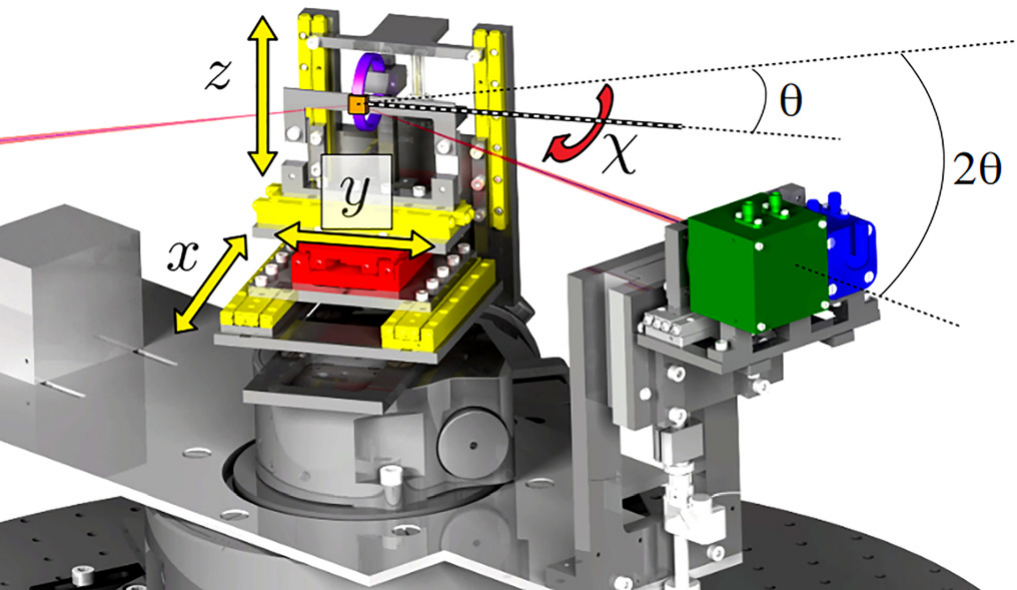
3D illustration of the instrument. The sample holder can be translated along three orthogonal directions (*x*, *y*, and *z*) (yellow arrows) and tilted by an angle *χ* (red). The electromagnet (violet) is placed close to the sample (orange). The avalanche photodiode (APD) (green) and the ultrafast photodiode (blue) are mounted on an arm describing a circular arc around the sample. The incidence angle at the sample, *θ*, is controlled by a further independent rotation.

Our time-resolved experiments were performed using a newly developed high-vacuum compatible reflectometer, illustrated in Fig. 3. The strong absorption of soft x rays under ambient conditions requires for the setup to be placed in a vacuum chamber ( $p≃1\times 10^{-7}\;\text{mbar}$). The sample holder and the detectors are mounted on in-vacuum stepper motors, which allow rotations in the horizontal plane by *θ* and 2θ, respectively. The sample holder can be moved in three orthogonal directions (*x*, *y*, and *z*, indicated in yellow) and an additional rotation enables us to tilt the sample surface with an angle *χ* (red). The translations and the rotations have, respectively, a resolution and reproducibility smaller than 180 nm and 0.01°. A home-built electromagnet was designed to reverse the magnetization of the magnetic thin films and is mounted next to the sample holder (purple). The magnet provides static magnetic fields up to 15 mT. The control of the motors and the collection of signals from the detectors are performed by a dedicated program developed in C. The code is optimized for the remote control of the setup and for the shot-by-shot recording of the incoming x-ray pulses.

As depicted in Fig. 1, both the infrared (IR) pump and the x-ray beams impinge on the sample surface at an angle *θ*, the reflectivity being probed in specular condition at an angle 2θ with respect to the incident direction. At the end of a 27 cm arm, two detectors are implemented: an avalanche photodiode (APD) used to record the intensity of reflected soft x rays and an ultrafast photodiode employed for the measurement of the IR reflectivity as for alignment and calibration. The angular aperture of the diodes was equal to 0.3° while the aperture of the beam on the diode was about 0.2°. To further play with the angular aperture, slits can be moved in front of the photodiodes to increase the angular selectivity. To prevent IR radiation from reaching the APD, a 400 nm thick aluminum filter covers the entrance of a protecting box enclosing the APD.

APDs are high-speed and high-sensitivity devices using a voltage named gain, to enhance their photo sensibility.^47^ The APD implemented in our setup (SAR3000e1, Laser Components) was benchmarked for readout speed with 707 eV photons at the Synchrotron SOLEIL. We found that its time resolution is around 4 ns, i.e., much shorter than the delay between subsequent x-ray pulses at FLASH2 (5 *μ*s; see Sec. II D). When increasing the gain voltage from 75 to 200 V, the output signal is amplified by 10 and is proportional to the light intensity. Note that by replacing the APD one dimension detector by a 2D one, our XRMR setup could take advantage of the low energy resolution^48^ or could be extended to x-ray resonant magnetic scattering, opening new opportunities to probe spin textures.^49,50^

### D. Pump–probe parameters and temporal structure

The TR-XRMR measurements were performed at the FL24 beamline of the FLASH2 FEL. The pump pulses were delivered by an IR laser ( λ=800 nm) with a pulse duration of 50 fs and linear polarization. The sample was probed by soft x-ray pulses tuned to the *L*_3_ edge of iron (see Sec. III A for more details on the probe energy) with an approximate duration of 80 fs and *π* polarization. The pump and probe beams reach the sample in a collinear geometry. The delay Δt separating the IR pulse from the x ray one can be varied up to several hundreds of picoseconds with an average temporal jitter of 100 fs.^51^ Note that in the future, the goal for FLASH2 is to achieve a jitter of 20 fs.

**FIG. 4.**
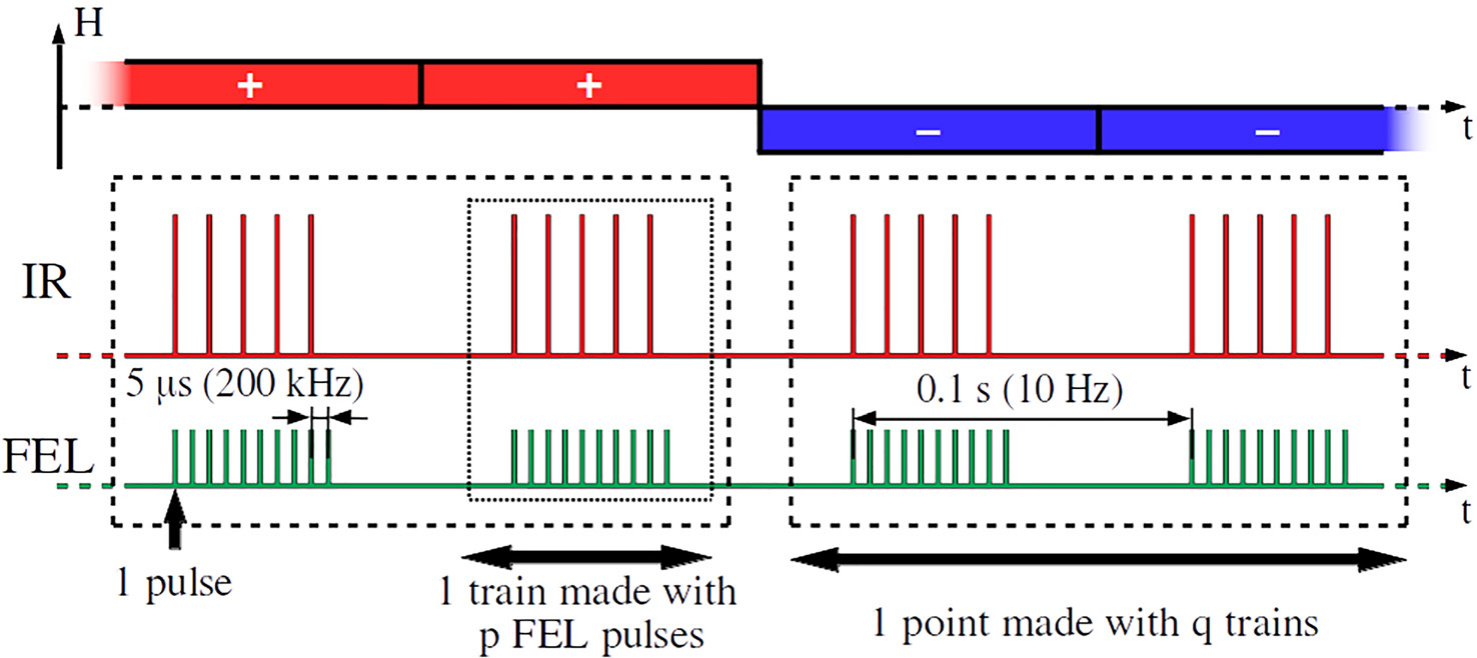
Temporal structure of the pump and probe pulses: each vertical line represents an IR (red) or x-ray (green) pulse. *p* is the number of pulses per train. *q* is the number of trains per data point. *H* is the magnetic field, which is reversed after *q* trains.

The radiation produced by FLASH2 is typically grouped in trains of pulses (Fig. 4) having a frequency of $f_{\mathrm{train}}=10\;\text{Hz}$. For our experiment, within a train, we had 40 pulses arriving at a frequency $f_{\text{FEL}}=200\;\text{kHz}$. As the FEL pulses result from a self-amplified spontaneous emission process, the intensity and the spectral distribution vary from pulse to pulse.^52^ Furthermore, the average light intensity can also fluctuate on longer timescales due to FEL instabilities.^53^ In order to compensate for these FEL fluctuations and improve the signal-to-noise ratio, we used a pump frequency equal to $f_{\text{FEL}}/2$ and averaged separately the “pumped” and the “unpumped” events over several trains. More precisely, we worked with 40 pulses per FEL train and used 10 trains for a set of constant measurement parameters, such as the angle *θ*, the delay Δt, and the magnetic field + or −. It is worth mentioning that the high pulse frequency of the FEL can induce significant static sample heating and even sample damage. With the aim of checking for such effects, we systematically compared the data points recorded at the beginning and at the end of a given pulse train and found no evidence for a change of the sample properties. This finding indicates that the sample has sufficient time to relax back to equilibrium between the pulses.

### E. Effective fluence

For angular scans, the variation of the incidence angle, *θ*, leads to an angle-dependent footprint of the pump and probe beams on the sample. At constant pulse intensity of the IR pump, this implies that the effective absorbed pump fluence also displays an angular dependence. This variation can be compensated by modulating the intensity of the incoming IR pulses, thanks to the combination of a wave plate (WP) and a polarizer. The effective fluence at the sample can be expressed as

$$F(\alpha_{\text{WP}},\theta ,S_{0})=E(\alpha_{\text{WP}})\frac{\;\text{sin}\;\theta }{S_{0}}\left[1-I_{\text{IR}}(\theta )\right],$$ (1)

where $E(\alpha_{\text{WP}})$ is the energy of the pump for a given WP angle $\alpha_{\text{WP}}$, *S*_0_ is the footprint of the beam on the sample at normal incidence, and $I_{\text{IR}}(\theta )$ stands for the IR reflectivity at a given incidence angle normalized between 0 and 1. We, therefore, measured $I_{\text{IR}}(\theta )$ as a function of the incidence angle *θ* and calculated the pump IR energy needed to get a constant absorbed fluence over a wide angular range. For practical reasons, at constant $E(\alpha_{\text{WP}})$, a variation of ±30% of the fluence was considered to be acceptable and angular scans were recorded over a reduced angular range. The data gathered for different angular ranges, each for a fixed $E(\alpha_{\text{WP}})$, were then stitched together to obtain the *θ*-scans shown in Sec. III. We set the effective pump fluence to be $1.6\pm 0.5\;{\text{mJcm}}^{-2}$ (corresponding to an average demagnetization of 25±7%) while the FEL fluence was $3.8\times 10^{-3}\;{\text{mJcm}}^{-2}$, well below the pumping threshold.^54^

## III. RESULTS AND DISCUSSION

The strategy for pump–probe measurements described above makes it possible to collect structural (*S*) and magnetic asymmetry (*A*) signals under pumped (Pu) and unpumped (Un) conditions. The structural (magnetic asymmetry) signal pumped and unpumped are, respectively, noted as $S_{\text{Pu}}$ ( $A_{\text{Pu}}$) and $S_{\text{Un}}$ ( $A_{\text{Un}}$). To highlight the effects of the pump on the structure and the magnetic asymmetry, we define the ratios $S_{n}=S_{\text{Pu}}/S_{\text{Un}}$ and $A_{n}=A_{\text{Pu}}/A_{\text{Un}}$ as normalized signals.

In the present study, we focused on two types of scans: angular scans where we vary the incident angle *θ* at a fixed delay Δt, and delay scans which record the entire time trace at one incidence angle *θ*. Before turning to the results, it is worthwhile examining the key points for the feasibility of such measurements at FLASH2.

### A. Soft x-ray photons with the FLASH2 third harmonic

**FIG. 5.**
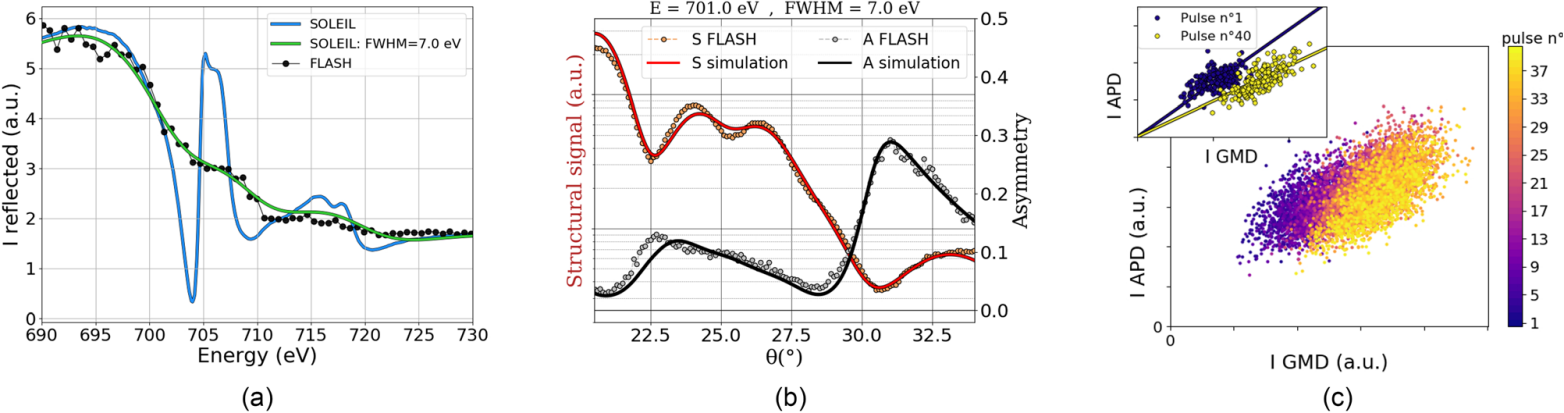
Static measurements. (a) Comparison of energy scans recorded at FLASH2 and at SOLEIL with an incident angle θ=7.5°. The data from FLASH2 (black dots), recorded with an energy resolution ΔE=7 eV, are compared to the results from SOLEIL (blue line, ΔE=140 meV). For the sake of a direct comparison, the FLASH data were compared with a scan of SOLEIL convoluted with a Gaussian profile having a full-width at half maximum (FWHM) of 7 eV (green line). (b) Unpumped structural *S* (orange dots) and magnetic asymmetry *A* (gray dots) signals recorded at FLASH2 with 701 eV photons. The simulations were made from the structural and magnetic parameters derived in the Table I, with the energy and resolution of FLASH2, namely, 701 ± 3.5 eV. (c) Correlation between the APD and GMD signals for θ=26.3°. The different colors correspond to the correlation for the *i*th pulse in the train. The inset shows only the correlations for the first (*i *=* *1) and the last pulse (*i *=* *40) in the train during 190 trains.

FLASH2 was designed for producing femtosecond light pulses with a fundamental wavelength down to 4 nm ( ≈300 eV).^55^ Such photon wavelengths are inadequate to probe the entire sample thickness with depth resolution below the nanometer range. However, when an FEL operates in the saturation regime, the microbunching of the electrons develops odd higher harmonics.^55–57^ One goal of the study was to examine the feasibility of using the third harmonic of the source to perform TR-XRMR studies at the Fe *L*_3_ edge. To prevent that the fundamental wavelength reaches the APD, one 400 nm Si and two 400 nm thick Al filters as well as a gas monitor detector (GMD) were used. With these devices, a ratio of 10^4^ between the intensity of the third and the first harmonic of the FEL can be estimated, which is sufficient to neglect the effects of the first order. To confirm this purpose, we have collected and compared static energy scans on the same sample at FLASH2 and at Synchrotron SOLEIL, as displayed in Fig. 5(a). The reflected intensities recorded at FLASH2 were taken with the third harmonic of a fundamental wavelength scanned around 5.26 nm, which was meant to deliver photons tuned to the *L*_3_ edge of iron. At these energies, there is no monochromator available on the FL24 beamline. The measurements shown in Fig. 5(a) for a fixed incidence angle θ=7.5° were performed at FLASH2 (black) and at SOLEIL (blue) and are both calibrated to the reference of Chen.^58^ Those two curves are quite different; however, if SOLEIL data are convoluted with a Gaussian profile having a full-width at half maximum (FWHM) of 7 eV, a good agreement is achieved. The result clearly confirms our ability to measure soft x rays around the Fe *L*_3_ edge at FLASH2 with a broad energy resolution of 7 eV.

In the rest of the study, we performed measurements at 701 eV because it is a good compromise to be as close as possible to the L-edge (706.8 eV based on the Ref. 58) while having a maximum of photons [which decrease with the photon energy, Fig. 5(a)]. Note that because of the broad energy resolution, even at 701 eV, we still get a good magnetic contrast as shown by the magnetic asymmetry in Fig. 5(b). In this figure, both structural signal and asymmetry were measured without pump and can be reproduced with the simulations using the structural and magnetic parameters obtained from the fit of angular scans carried out at SOLEIL (Fig. 2 and Table I), by only changing the energy resolution to 7 eV FWHM. This underlines the feasibility of XRMR measurements with the third harmonic at FLASH2.

### B. Detector linearity

Because of the FEL intensity fluctuations already discussed in Sec. II D, it turns indispensable to normalize the probed reflectivity by the incoming FEL intensity.^59,60^ For that, we used the FEL intensity measured by upsteam GMD available at the FL24 beamline,^57^ which has the advantage of delivering information on individual x-ray pulses. As this upstream GMD is placed before the different filters used to attenuate the first radiation harmonic, both the first and the third harmonics are simultaneously monitored. As a reminder, the reflectivity recorded by our APD is only coming from the third harmonic (see Sec. III A).

Figure 5(c) shows the correlation between the intensities measured pulse-by-pulse by the GMD and the APD at constant *θ*, Δt and applied magnetic field. It is obvious that the correlation between the GMD and the APD changes during the train: over the first pulses, the correlation is rather linear (see blue curve in the inset) and gradually evolves toward a better linearity with a different slope for the last pulse.^61^ This observation clearly indicates that it is necessary to use a pulse-by-pulse normalization of the measured intensity.

### C. Reproducibility of the data

**FIG. 6.**
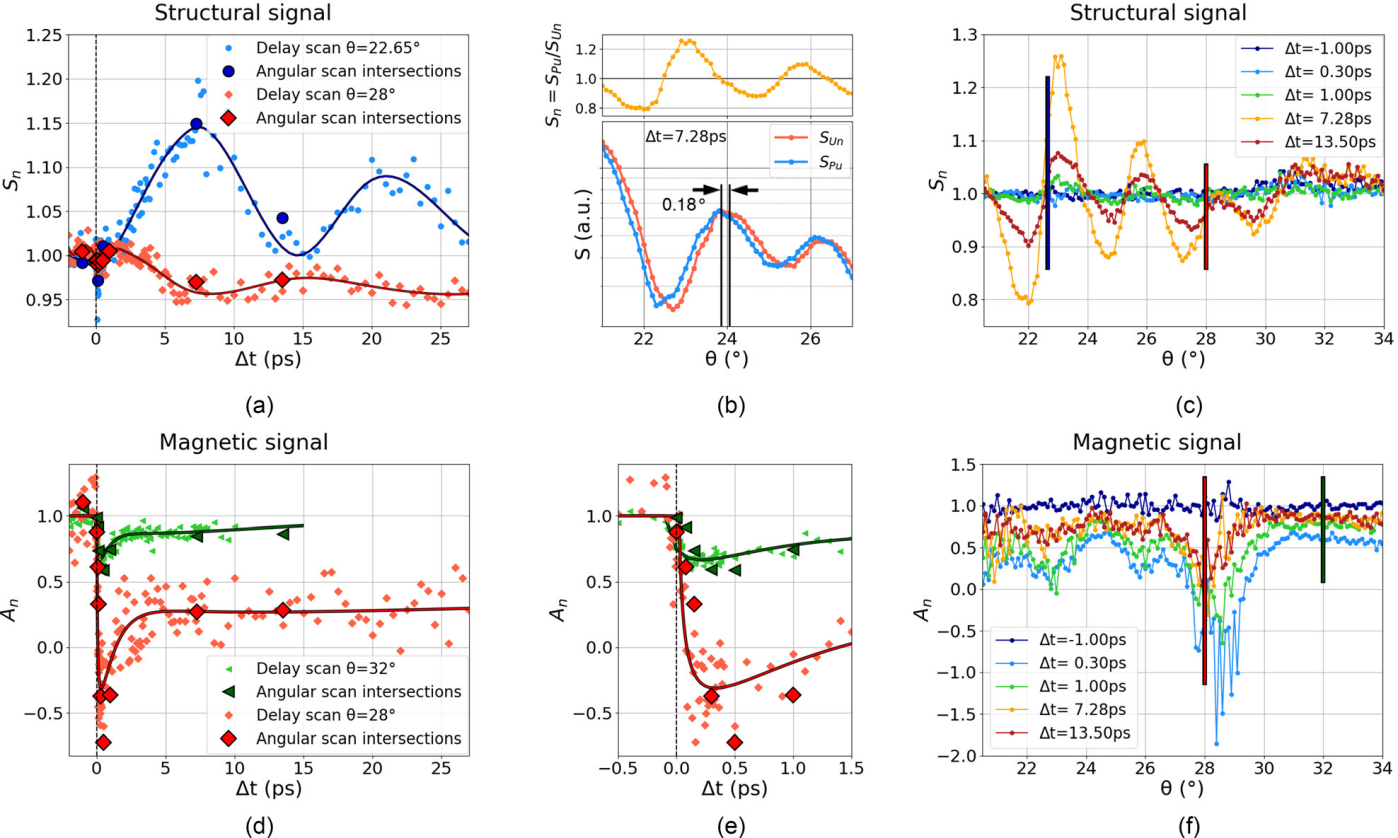
Time resolved measurements. (a) Delay scan displaying the variation of the normalized structural signal *S_n_* (small dots) as a function of the delay at two incidence angles. Solid lines are guides to the eye. (b) For Δt=7.28 ps, the bottom subfigure shows $S_{\text{Pu}}$ and $S_{\text{Un}}$, respectively, the pumped and unpumped experimental structural data, while the top subfigure displays their ratio $S_{n}=S_{\text{Pu}}/S_{\text{Un}}$ (c) Angular scans of the normalized structural signal *S_n_* for various delays. The two vertical blue and red solid lines in panel (c), located at θ=22.65° and 28°, indicate the selected *S_n_* values shown with large symbols of the same color in panel (a). Lower panels (d) and (f) follow a similar illustration strategy than (a) and (c) but display delay and angular scan results for the magnetic asymmetry signal *A_n_*. (e) Same as (d) but zoomed on early time delays. All data were recorded with 701 ± 3.5 eV photons.

In order to prove the robustness of our measurements, we cross-checked data recorded under the same conditions but in two different manners: angular and delay scans. Typical TR-XRMR results obtained at FLASH2 are shown in Fig. 6. The figure displays the structural signals *S_n_* (upper panels) and the magnetic asymmetry signals *A_n_* (lower panels) recorded during delay scans (left panels) and angular scans (right panels). For comparison, selected data points from the angular scans (right panels) are drawn with large symbols on top of the delay scans (left panels). The juxtaposition clearly indicates that the measured values are independent of the type of scan and demonstrates the reliability of the experimental setup.

### D. Ultrafast magnetic and structural dynamics

The *A_n_* and *S_n_* responses illustrated in Fig. 6 deliver sharp insight into the evolution of magnetic and structural properties of the iron film following the pump excitation. The angular scans in Figs. 6(c) and 6(f), respectively, display large contrast for different delays Δt>0.

The magnetic asymmetry signal *A_n_*, Figs. 6(d) and 6(e), decreases quasi instantly after the pump, reaches a minimum at 0.3 ps, and recovers slowly for longer delays. This behavior matches the expected ultrafast demagnetization of the iron film and gives a time constant of the ultrafast demagnetization τ=90±40 fs. Note that *A_n_* is not proportional to the bare magnetization but incorporates the detailed magnetic depth profile of the sample, which can lead to values outside the [0,1] interval. In contrast, the structural signal *S_n_* in Figs. 6(a) and 6(c) stays rather constant for delays Δt<1 ps and changes to reach a maximum amplitude at 7.28 ps, for both incidence angles. The constant *S_n_* signal before 1ps implies that for these timescales, the *A_n_* changes are purely driven by magnetization changes. For longer delays, *S_n_* displays a damped oscillating behavior, with a period roughly equal to 2 × 7.28 ps. This characteristic delay corresponds to the time needed for an acoustic sound wave to pass through all layers from the surface to the substrate at the speed of sound. This result is in agreement with recent observations^32,62–65^ concluding that the ultrafast demagnetization process is accompanied by a strain wave that expands with a velocity of a few nm ps^−1^.

The angular scans are currently quantitatively analyzed using the matrix formalism implemented in the DYNA code (see Sec. II A). The detailed aspects of the numerical treatment are beyond the scope of this paper and will be addressed separately. As anticipated, the reflectivity curves can be fitted to extract transient parameters like the thicknesses and the magnetic profile of the layers. More easily, the oscillations of *S_n_* shown in Fig. 6(c) can already be related to a dilation of the layer; a shift of 0.18° between the two curves can be observed in Fig. 6(b) displaying the structural unpumped $S_{\text{Un}}$ and pumped signals $S_{\text{Pu}}$. From this shift and with the Bragg's law, it is possible to determine the thickness *d* of the iron film^37,66^ and the dilation of the film upon laser excitation $d_{\text{Pu}}-d_{\text{Un}}$. By considering only the Fe layer, we discern a dilation $d_{\text{Pu}}-d_{\text{Un}}$ of ≈1.5 Å. A more precise value can be derived when accounting for all layers of the sample, a task which is rather straightforward with DYNA, and gives a dilation of ≈2 Å. A more quantitative analysis will allow us to retrieve exactly from which layers this transient dilatation is coming from.

**FIG. 7.**
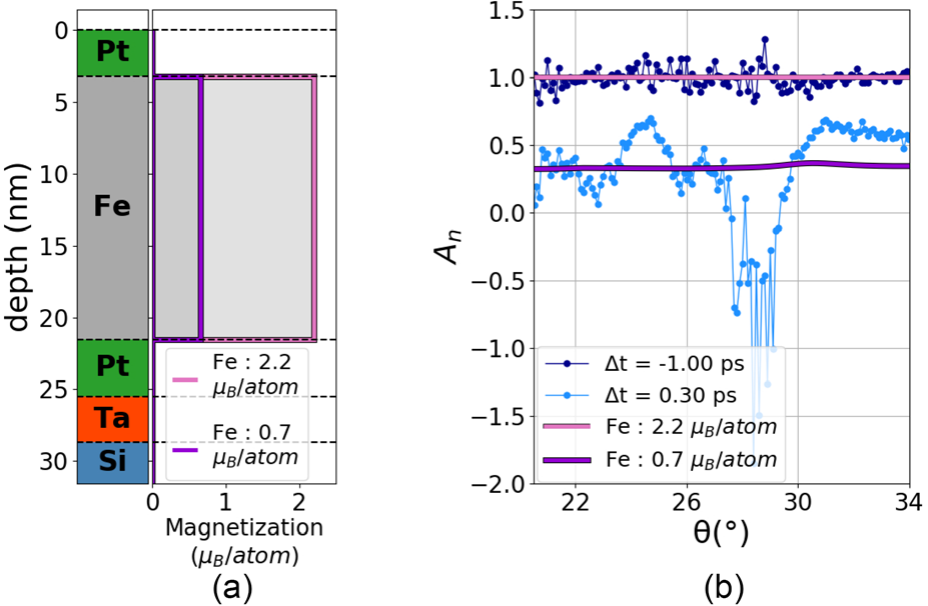
Homogeneous demagnetization: (a) representation of the static sample structure and of two magnetic depth profiles. The different layers of the static sample structure are illustrated by bands of different colors, whereas the two homogeneous magnetic profiles are drawn by two lines as a function of the depth. The magnetization for these magnetic profiles is constant in the iron [ $2.2\;\mu_{B}$/atom (pink line) and $0.7\;\mu_{B}$/atom (purples line)], with *μ_B_* the Bohr magneton and null for the other layers. (b) Comparison of the normalized magnetic asymmetry signals *A_n_* for the two measurements performed at FLASH2 and for the two simulations. The measurements are angular scans at two different delays [ Δt=−1.00 ps (darkblue dots) and Δt=0.30 ps (lightblue dots)], while the two simulations of *A_n_* are those of the magnetic profiles illustrated in (a) [Fe magnetization equal to $2.2\;\mu_{B}$/atom (pink line) and $0.7\;\mu_{B}$/atom (purple line)].

The main added value of TR-XRMR experiments is the potential for determining the evolution of the magnetic depth profile with time. While the sample considered in this contribution was merely designed for the benchmark of TR-XRMR experiments at FLASH2, we can already put forward an important finding: the results rule out the scenario of a homogeneous demagnetization. This finding is illustrated in Fig. 7, which shows simulations of *A_n_* with a homogeneous demagnetization imposed through the Fe layer. In other words, as shown on the left, the static magnetic moment of the entire layer is assumed to be simply reduced during the demagnetization process. In this case, the simulations show that the *A_n_* signal should display almost no angular dependence, which is in contradiction to the measured data displayed in Fig. 6(f) and also shown in Fig. 7 for Δt of 300 fs. While further quantitative analysis is needed to extract the transient depth magnetic profile of the iron layer, it becomes clear that the present results call into question the scenario of homogeneous demagnetization. In line with our observation, recent studies by Chen *et al.*^17,18^ and Shokeen *et al.*^9^ reported on dissimilarities between the demagnetization of the front and back layers of ferromagnetic layers. A more quantitative analysis, which is currently ongoing, will reveal the precise transient depth magnetic and structural profile, bringing new information to understand the complex femtomagnetism processes.

## IV. CONCLUSION

In this article, we demonstrate the feasibility of TR-XRMR measurements at FLASH2 with photons tuned to the Fe *L*_3_-edge. While FLASH2 was designed for optimal performance for energies up to 310 eV, we found that properties of the third harmonic are adequate for TR-XRMR studies at higher energies. Despite a broad energy resolution of 7 eV, FLASH2 measurements are successfully confronted with a set of reference data collected at Synchrotron SOLEIL. We review key experimental aspects and demonstrate how to extract the best magnetic and structural responses of the sample. The TR-XRMR highlights two different dynamics at different time scales. Within the first hundreds of femtosecond, the magnetic asymmetry signal shows an ultrafast decrease linked to the usual ultrafast demagnetization while the structural signals begin to change after a few picoseconds. Our preliminary analysis highlights the non-homogeneity of the demagnetization in depth as well as a dilation and oscillation of the thin film thickness due to an optically launched strain wave. This work demonstrates the potential of TR-XRMR performed at FEL to unravel what are the microscopic mechanisms at play after an ultrafast optical excitation of a magnetic thin film.

## Data Availability

The data that support the findings of this study are available from the corresponding author upon reasonable request.

## References

[1] A. Kirilyuk , A. V. Kimel , and T. Rasing , Rev. Mod. Phys. 82, 2731 (2010). 10.1103/RevModPhys.82.2731

[2] E. Beaurepaire , J.-C. Merle , A. Daunois , and J.-Y. Bigot , Phys. Rev. Lett. 76, 4250 (1996). 10.1103/PhysRevLett.76.4250 10061239

[3] M. Hennecke , I. Radu , R. Abrudan , T. Kachel , K. Holldack , R. Mitzner , A. Tsukamoto , and S. Eisebitt , Phys. Rev. Lett. 122, 157202 (2019). 10.1103/PhysRevLett.122.157202 31050542

[4] S. Jana , R. S. Malik , Y. O. Kvashnin , I. L. M. Locht , R. Knut , R. Stefanuik , I. D. Marco , A. N. Yaresko , M. Ahlberg , J. Åkerman , R. Chimata , M. Battiato , J. Söderström , O. Eriksson , and O. Karis , Phys. Rev. Res. 2, 013180 (2020). 10.1103/PhysRevResearch.2.013180

[5] C. D. Stanciu , F. Hansteen , A. V. Kimel , A. Kirilyuk , A. Tsukamoto , A. Itoh , and T. Rasing , Phys. Rev. Lett. 99, 047601 (2007). 10.1103/PhysRevLett.99.047601 17678404

[6] G.-M. Choi , B.-C. Min , K.-J. Lee , and D. G. Cahill , Nat. Commun. 5, 4334 (2014). 10.1038/ncomms5334 25007978

[7] B. Koopmans , G. Malinowski , F. Dalla Longa , D. Steiauf , M. Fähnle , T. Roth , M. Cinchetti , and M. Aeschlimann , Nat. Mater. 9, 259 (2010). 10.1038/nmat2593 20010830

[8] M. Battiato , K. Carva , and P. M. Oppeneer , Phys. Rev. Lett. 105, 027203 (2010). 10.1103/PhysRevLett.105.027203 20867735

[9] V. Shokeen , M. Sanchez Piaia , J.-Y. Bigot , T. Müller , P. Elliott , J. K. Dewhurst , S. Sharma , and E. K. U. Gross , Phys. Rev. Lett. 119, 107203 (2017). 10.1103/PhysRevLett.119.107203 28949167

[10] W. Zhang , W. He , X.-Q. Zhang , Z.-H. Cheng , J. Teng , and M. Fähnle , Phys. Rev. B 96, 220415 (2017). 10.1103/PhysRevB.96.220415

[11] M. Malvestuto , R. Ciprian , A. Caretta , B. Casarin , and F. Parmigiani , J. Phys.: Condens. Matter 30, 053002 (2018). 10.1088/1361-648X/aaa211 29315080

[12] B. Vodungbo , J. Gautier , G. Lambert , A. B. Sardinha , M. Lozano , S. Sebban , M. Ducousso , W. Boutu , K. Li , B. Tudu , M. Tortarolo , R. Hawaldar , R. Delaunay , V. López-Flores , J. Arabski , C. Boeglin , H. Merdji , P. Zeitoun , and J. Lüning , Nat. Commun. 3, 999 (2012). 10.1038/ncomms2007 22893123

[13] D. Rudolf , C. La-O-Vorakiat , M. Battiato , R. Adam , J. M. Shaw , E. Turgut , P. Maldonado , S. Mathias , P. Grychtol , H. T. Nembach , T. J. Silva , M. Aeschlimann , H. C. Kapteyn , M. M. Murnane , C. M. Schneider , and P. M. Oppeneer , Nat. Commun. 3, 1037 (2012). 10.1038/ncomms2029 22948819

[14] E. Turgut , C. La-O-Vorakiat , J. M. Shaw , P. Grychtol , H. T. Nembach , D. Rudolf , R. Adam , M. Aeschlimann , C. M. Schneider , T. J. Silva , M. M. Murnane , H. C. Kapteyn , and S. Mathias , Phys. Rev. Lett. 110, 197201 (2013). 10.1103/PhysRevLett.110.197201 23705737

[15] M. Hofherr , P. Maldonado , O. Schmitt , M. Berritta , U. Bierbrauer , S. Sadashivaiah , A. J. Schellekens , B. Koopmans , D. Steil , M. Cinchetti , B. Stadtmüller , P. M. Oppeneer , S. Mathias , and M. Aeschlimann , Phys. Rev. B 96, 100403 (2017). 10.1103/PhysRevB.96.100403

[16] A. Alekhin , I. Razdolski , N. Ilin , J. P. Meyburg , D. Diesing , V. Roddatis , I. Rungger , M. Stamenova , S. Sanvito , U. Bovensiepen , and A. Melnikov , Phys. Rev. Lett. 119, 017202 (2017). 10.1103/PhysRevLett.119.017202 28731774

[17] J. Chen , J. Wieczorek , A. Eschenlohr , S. Xiao , A. Tarasevitch , and U. Bovensiepen , Appl. Phys. Lett. 110, 092407 (2017). 10.1063/1.4977767

[18] J. Chen , U. Bovensiepen , A. Eschenlohr , T. Müller , P. Elliott , E. K. U. Gross , J. K. Dewhurst , and S. Sharma , Phys. Rev. Lett. 122, 067202 (2019). 10.1103/PhysRevLett.122.067202 30822073

[19] R.-P. Pan , H. D. Wei , and Y. R. Shen , Phys. Rev. B 39, 1229 (1989). 10.1103/PhysRevB.39.1229 9948307

[20] B. Pfau , S. Schaffert , L. Müller , C. Gutt , A. Al-Shemmary , F. Büttner , R. Delaunay , S. Düsterer , S. Flewett , R. Frömter , J. Geilhufe , E. Guehrs , C. M. Günther , R. Hawaldar , M. Hille , N. Jaouen , A. Kobs , K. Li , J. Mohanty , H. Redlin , W. F. Schlotter , D. Stickler , R. Treusch , B. Vodungbo , M. Kläui , H. P. Oepen , J. Lüning , G. Grübel , and S. Eisebitt , Nat. Commun. 3, 1100 (2012). 10.1038/ncomms2108 23033076 PMC3493637

[21] G. Fan , K. Légaré , V. Cardin , X. Xie , E. Kaksis , G. Andriukaitis , A. Pugžlys , B. E. Schmidt , J. P. Wolf , M. Hehn , G. Malinowski , B. Vodungbo , E. Jal , J. Lüning , N. Jaouen , Z. Tao , A. Baltuška , F. Légaré , and T. Balčiun̄as , “ Time-resolving magnetic scattering on rare-earth ferrimagnets with a bright soft-X-ray high-harmonic source,” arXiv:1910.14263 (2019).

[22] D. Zusin , E. Iacocca , L. L. Guyader , A. H. Reid , W. F. Schlotter , T.-M. Liu , D. J. Higley , G. Coslovich , S. F. Wandel , P. M. Tengdin , S. K. K. Patel , A. Shabalin , N. Hua , S. B. Hrkac , H. T. Nembach , J. M. Shaw , S. A. Montoya , A. Blonsky , C. Gentry , M. A. Hoefer , M. M. Murnane , H. C. Kapteyn , E. E. Fullerton , O. Shpyrko , H. A. Dürr , and T. J. Silva , “ Ultrafast domain dilation induced by optical pumping in ferromagnetic CoFe/Ni multilayers,” arXiv:2001.11719 (2020).

[23] M. Hennes , A. Merhe , X. Liu , D. Weder , C. v K. Schmising , M. Schneider , C. M. Günther , B. Mahieu , G. Malinowski , M. Hehn , D. Lacour , F. Capotondi , E. Pedersoli , I. P. Nikolov , V. Chardonnet , E. Jal , J. Lüning , and B. Vodungbo , Phys. Rev. B 102, 174437 (2020). 10.1103/PhysRevB.102.174437

[24] T. Sant , D. Ksenzov , F. Capotondi , E. Pedersoli , M. Manfredda , M. Kiskinova , H. Zabel , M. Kläui , J. Lüning , U. Pietsch , and C. Gutt , Sci. Rep. 7, 15064 (2017). 10.1038/s41598-017-15234-7 29118451 PMC5678147

[25] J. Wieczorek , A. Eschenlohr , B. Weidtmann , M. Rösner , N. Bergeard , A. Tarasevitch , T. O. Wehling , and U. Bovensiepen , Phys. Rev. B 92, 174410 (2015). 10.1103/PhysRevB.92.174410

[26] U. Bierbrauer , S. T. Weber , D. Schummer , M. Barkowski , A.-K. Mahro , S. Mathias , H. C. Schneider , B. Stadtmüller , M. Aeschlimann , and B. Rethfeld , J. Phys.: Condens. Matter 29, 244002 (2017). 10.1088/1361-648X/aa6f73 28510535

[27] W. You , P. Tengdin , C. Chen , X. Shi , D. Zusin , Y. Zhang , C. Gentry , A. Blonsky , M. Keller , P. M. Oppeneer , H. Kapteyn , Z. Tao , and M. Murnane , Phys. Rev. Lett. 121, 077204 (2018). 10.1103/PhysRevLett.121.077204 30169091

[28] M. Elyasi and H. Yang , Phys. Rev. B 94, 024417 (2016). 10.1103/PhysRevB.94.024417

[29] W.-T. Lu , Y. Zhao , M. Battiato , Y. Wu , and Z. Yuan , Phys. Rev. B 101, 014435 (2020). 10.1103/PhysRevB.101.014435

[30] E. Jal , M. Dabrowski , J.-M. Tonnerre , M. Przybylski , S. Grenier , N. Jaouen , and J. Kirschner , Phys. Rev. B 87, 224418 (2013). 10.1103/PhysRevB.87.224418

[31] E. Jal , J. B. Kortright , T. Chase , T. Liu , A. X. Gray , P. Shafer , E. Arenholz , P. Xu , J. Jeong , M. G. Samant , S. S. P. Parkin , and H. A. Dürr , Appl. Phys. Lett. 107, 092404 (2015). 10.1063/1.4929990

[32] E. Jal , V. López-Flores , N. Pontius , T. Ferté , N. Bergeard , C. Boeglin , B. Vodungbo , J. Lüning , and N. Jaouen , Phys. Rev. B 95, 184422 (2017). 10.1103/PhysRevB.95.184422

[33] C. Gutt , T. Sant , D. Ksenzov , F. Capotondi , E. Pedersoli , L. Raimondi , I. P. Nikolov , M. Kiskinova , S. Jaiswal , G. Jakob , M. Kläui , H. Zabel , and U. Pietsch , Struct. Dyn. 4, 055101 (2017). 10.1063/1.4990650 28713843 PMC5500121

[34] M. Elzo , E. Jal , O. Bunau , S. Grenier , Y. Joly , A. Ramos , H. Tolentino , J. Tonnerre , and N. Jaouen , J. Magn. Magn. Mater. 324, 105 (2012). 10.1016/j.jmmm.2011.07.019

[35] We used the DYNA package, freely downloadable at http://dyna.neel.cnrs.fr/

[36] B. Vidal and P. Vincent , Appl. Opt. 23, 1794 (1984). 10.1364/AO.23.001794 18212906

[37] S. Macke and E. Goering , J. Phys.: Condens. Matter 26, 363201 (2014). 10.1088/0953-8984/26/36/363201 25121937

[38] J. Stöhr and H. C. Siegmann , *Solid-State Sciences* ( Springer, Berlin/Heidelberg, 2006), Vol. 5.

[39] J. B. Kortright , J. Electron. Spectrosc. Relat. Phenom. 189, 178 (2013). 10.1016/j.elspec.2013.01.019

[40] S. Brück , “ Magnetic resonant reflectometry on exchange bias systems,” Ph.D. thesis ( University of Stuttgart, 2009).

[41] H.-C. Mertins , D. Abramsohn , A. Gaupp , F. Schäfers , W. Gudat , O. Zaharko , H. Grimmer , and P. M. Oppeneer , Phys. Rev. B 66, 184404 (2002). 10.1103/PhysRevB.66.184404

[42] J.-M. Tonnerre , E. Jal , E. Bontempi , N. Jaouen , M. Elzo , S. Grenier , H. L. Meyerheim , and M. Przybylski , Eur. Phys. J.: Spec. Top. 208, 177 (2012). 10.1140/epjst/e2012-01618-y

[43] As shown by Kortright,^39^ the resonant magnetic x-ray reflectivity is proportional to $C^{2}\pm CM$, where *C* indicates the pure charge contribution and *M* the pure magnetic contribution. Note that *M*^2^ is not considered and thus omitted. The non-magnetic contribution $I_{\text{NM}}\;{=}^{.}S=(I^{+}+I^{-})/2$ is therefore proportional to *C*^2^ and the magnetic asymmetry $A=(I^{+}-I^{-})/(I^{+}+I^{-})$ is proportional to *M*/*C*. The pure magnetic contribution is therefore given as $M=A\sqrt{I_{\text{NM}}}$.

[44] N. Jaouen , J.-M. Tonnerre , G. Kapoujian , P. Taunier , J.-P. Roux , D. Raoux , and F. Sirotti , J. Synchrotron Radiat. 11, 353 (2004). 10.1107/S0909049504013767 15211043

[45] M. Sacchi , N. Jaouen , H. Popescu , R. Gaudemer , J. M. Tonnerre , S. G. Chiuzbaian , C. F. Hague , A. Delmotte , J. M. Dubuisson , G. Cauchon , B. Lagarde , and F. Polack , J. Phys. Conf. Ser. 425, 072018 (2013). 10.1088/1742-6596/425/7/072018

[46] C. Klewe , T. Kuschel , J.-M. Schmalhorst , F. Bertram , O. Kuschel , J. Wollschläger , J. Strempfer , M. Meinert , and G. Reiss , Phys. Rev. B 93, 214440 (2016). 10.1103/PhysRevB.93.214440 26371679

[47] T. Kaneda , *Lightwave Communications Technology*, Semiconductors and Semimetals Vol. 22, Part D, edited by W. T. Tsang ( Elsevier, New York, 1985), Chap. 3, p. 247.

[48] R. Y. Engel , P. S. Miedema , D. Turenne , I. Vaskivskyi , G. Brenner , S. Dziarzhytski , M. Kuhlmann , J. O. Schunck , F. Döring , A. Styervoyedov , S. S. Parkin , C. David , C. Schüßler-Langeheine , H. A. Dürr , and M. Beye , Appl. Sci. 10, 6947 (2020). 10.3390/app10196947

[49] J.-Y. Chauleau , W. Legrand , N. Reyren , D. Maccariello , S. Collin , H. Popescu , K. Bouzehouane , V. Cros , N. Jaouen , and A. Fert , Phys. Rev. Lett. 120, 037202 (2018). 10.1103/PhysRevLett.120.037202 29400492

[50] C. Leveille , E. Burgos-Parra , Y. Sassi , F. Ajejas , V. Chardonnet , E. Pedersoli , F. Capotondi , G. D. Ninno , F. Maccherozzi , S. Dhesi , D. M. Burn , G. van der Laan , O. S. Latcham , A. V. Shytov , V. V. Kruglyak , E. Jal , V. Cros , J.-Y. Chauleau , N. Reyren , M. Viret , and N. Jaouen , “ Ultrafast time-evolution of chiral Néel magnetic domain walls probed by circular dichroism in x-ray resonant magnetic scattering,” arXiv:2007.08583 [cond-mat.mtrl-sci] (2021). 10.1038/s41467-022-28899-0 PMC8931105 35301298

[51] F. Lever , D. Mayer , D. Picconi , J. Metje , S. Alisauskas , F. Calegari , S. Düsterer , C. Ehlert , R. Feifel , M. Niebuhr , B. Manschwetus , M. Kuhlmann , T. Mazza , M. S. Robinson , R. J. Squibb , A. Trabattoni , M. Wallner , P. Saalfrank , T. J. A. Wolf , and M. Gühr , J. Phys. B 54, 014002 (2020). 10.1088/1361-6455/abc9cb

[52] K. Tiedtke , A. Azima , N. von Bargen , L. Bittner , S. Bonfigt , S. Düsterer , B. Faatz , U. Frühling , M. Gensch , C. Gerth , N. Guerassimova , U. Hahn , T. Hans , M. Hesse , K. Honkavaar , U. Jastrow , P. Juranic , S. Kapitzki , B. Keitel , T. Kracht , M. Kuhlmann , W. B. Li , M. Martins , T. Núñez , E. Plönjes , H. Redlin , E. L. Saldin , E. A. Schneidmiller , J. R. Schneider , S. Schreiber , N. Stojanovic , F. Tavella , S. Toleikis , R. Treusch , H. Weigelt , M. Wellhöfer , H. Wabnitz , M. V. Yurkov , and J. Feldhaus , New J. Phys. 11, 023029 (2009). 10.1088/1367-2630/11/2/023029

[53] E. L. Saldin , E. A. Schneidmiller , and M. V. Yurkov , New J. Phys. 12, 035010 (2010). 10.1088/1367-2630/12/3/035010

[54] T. Wang , D. Zhu , B. Wu , C. Graves , S. Schaffert , T. Rander , L. Müller , B. Vodungbo , C. Baumier , D. P. Bernstein , B. Bräuer , V. Cros , S. de Jong , R. Delaunay , A. Fognini , R. Kukreja , S. Lee , V. López-Flores , J. Mohanty , B. Pfau , H. Popescu , M. Sacchi , A. B. Sardinha , F. Sirotti , P. Zeitoun , M. Messerschmidt , J. J. Turner , W. F. Schlotter , O. Hellwig , R. Mattana , N. Jaouen , F. Fortuna , Y. Acremann , C. Gutt , H. A. Dürr , E. Beaurepaire , C. Boeglin , S. Eisebitt , G. Grübel , J. Lüning , J. Stöhr , and A. O. Scherz , Phys. Rev. Lett. 108, 267403 (2012). 10.1103/PhysRevLett.108.267403 23005013

[55] S. Schreiber and B. Faatz , High Power Laser Sci. Eng. 3, e20 (2015). 10.1017/hpl.2015.16

[56] P. Schmüser , M. Dohlus , J. Rossbach , and C. Behrens , *Free-Electron Lasers in the Ultraviolet and X-Ray Regime* ( Springer, 2014), Vol. 258, pp. 13–20.

[57] J. Rossbach , J. R. Schneider , and W. Wurth , Phys. Rep. 808, 1 (2019). 10.1016/j.physrep.2019.02.002

[58] C. T. Chen , Y. U. Idzerda , H.-J. Lin , N. V. Smith , G. Meigs , E. Chaban , G. H. Ho , E. Pellegrin , and F. Sette , Phys. Rev. Lett. 75, 152 (1995). 10.1103/PhysRevLett.75.152 10059138

[59] D. J. Higley , K. Hirsch , G. L. Dakovski , E. Jal , E. Yuan , T. Liu , A. A. Lutman , J. P. MacArthur , E. Arenholz , Z. Chen , G. Coslovich , P. Denes , P. W. Granitzka , P. Hart , M. C. Hoffmann , J. Joseph , L. L. Guyader , A. Mitra , S. Moeller , H. Ohldag , M. Seaberg , P. Shafer , J. Stöhr , A. Tsukamoto , H.-D. Nuhn , A. H. Reid , H. A. Dürr , and W. F. Schlotter , Rev. Sci. Instrum. 87, 033110 (2016). 10.1063/1.4944410 27036761

[60] K. Tiedtke , A. A. Sorokin , U. Jastrow , P. Juranić , S. Kreis , N. Gerken , M. Richter , U. Arp , Y. Feng , D. Nordlund , R. Soufli , M. Fernández-Perea , L. Juha , P. Heimann , B. Nagler , H. J. Lee , S. Mack , M. Cammarata , O. Krupin , M. Messerschmidt , M. Holmes , M. Rowen , W. Schlotter , S. Moeller , and J. J. Turner , Opt. Express 22, 21214 (2014). 10.1364/OE.22.021214 25321502

[61] The origin of the changes in linearity is not fully understood, but our best hypothesis is (i) that over a train, there is a small drift of the pointing of the x-ray; (ii) the first pulse gives a different GMD intensity because there are no charge effects of the previous pulses.

[62] T. Henighan , M. Trigo , S. Bonetti , P. Granitzka , D. Higley , Z. Chen , M. P. Jiang , R. Kukreja , A. Gray , A. H. Reid , E. Jal , M. C. Hoffmann , M. Kozina , S. Song , M. Chollet , D. Zhu , P. F. Xu , J. Jeong , K. Carva , P. Maldonado , P. M. Oppeneer , M. G. Samant , S. S. P. Parkin , D. A. Reis , and H. A. Dürr , Phys. Rev. B 93, 220301 (2016). 10.1103/PhysRevB.93.220301

[63] A. von Reppert , L. Willig , J.-E. Pudell , M. Rössle , W. Leitenberger , M. Herzog , F. Ganss , O. Hellwig , and M. Bargheer , Appl. Phys. Lett. 113, 123101 (2018). 10.1063/1.5050234

[64] D. Schick , M. Herzog , A. Bojahr , W. Leitenberger , A. Hertwig , R. Shayduk , and M. Bargheer , Struct. Dyn. 1, 064501 (2014). 10.1063/1.4901228 26798784 PMC4714650

[65] J. Pudell , A. von Reppert , D. Schick , F. Zamponi , M. Rössle , M. Herzog , H. Zabel , and M. Bargheer , Phys. Rev. B 99, 094304 (2019). 10.1103/PhysRevB.99.094304

[66] The structural signal of the Fe layer shows several oscillations (see Fig. 2), the first one at $\theta_{1}=5.00{}^{\circ}$ and the seventh $\theta_{7,\text{Un}}=24.04{}^{\circ}$ or $\theta_{8,\text{Pu}}=23.86{}^{\circ}$ for the unpumped and the pumped curves [see Fig. 6(b)]. With a photon energy of 701eV ( λ=1.768 nm) and an average angular difference between the peak due to the iron layer $\Delta \theta =(\theta_{7}-\theta_{1})/6$, the thickness *d* of the iron layer can be estimated by $d=(\lambda /2){\left[\text{sin}\;(\theta_{1}+\Delta \theta )-\text{sin}\;\theta_{1}\right]}^{-1}$.

